# Rootstock–scion interaction affects *Malus* transcriptome profiles in response to cadmium

**DOI:** 10.1038/s41597-023-02239-3

**Published:** 2023-05-23

**Authors:** Yijin Huang, Luyang Sun, Jiale Wang, Yahui Chen, Jiali He, Deguo Lyu

**Affiliations:** 1 College of Horticulture, Shenyang Agricultural University, Shenyang, Liaoning 110866 China; 2 Key Lab of Fruit Quality Development and Regulation of Liaoning Province, Shenyang, Liaoning 110866 China

**Keywords:** Abiotic, Plant physiology

## Abstract

Apple production is threatened by cadmium contamination in orchards. Cd accumulation and tolerance in grafted *Malus* plants is affected by rootstock, scion, and their interaction. This dataset is part of an experiment investigating the molecular mechanism of Cd bioaccumulation and tolerance in different apple rootstock-scion combinations. We exposed four rootstock–scion combinations to Cd treatment consisting of Hanfu and Fuji apple (*Malus domestica*) scions grafted onto apple rootstocks of *M. baccata* or *M. micromalus* “qingzhoulinqin”. RNA sequencing was conducted in roots and leaves of grafting combinations under 0 or 50 μM CdCl_2_ conditions. A comprehensive transcriptional dataset of affected rootstock, scion, and their interaction among different graft combinations was obtained. This dataset provides new insights in the transcriptional control of Cd bioaccumulation and tolerance in grafting plants regulated by rootstock and scion. Herein, we discuss the molecular mechanism underlying Cd absorption and bioaccumulation.

## Background & Summary

Industrial activity and agrochemical overuse, especially metal-based pesticide and fertiliser application, lead to cadmium (Cd) bioaccumulation in orchards to varying degrees^1,2^. For example, in the Liaodong Peninsula, a major apple production area in Northern China, 6.34% of the fruit peel samples exceeded the allowable levels of Cd (0.03 mg kg^−1^, dry weight)^3^. The Cd content of oranges, grapes, pears, and plums also exceeded the safety standard in some orchards in Zhejiang Province^4^. When absorbed excessively by plants, Cd may cause many physiological, biochemical, and cellular changes^5^. Excess Cd levels can inhibit net photosynthesis, diminish chlorophyll biosynthesis, reduce the absorption of mineral elements, break the balance between reactive oxygen species (ROS) and antioxidants, induce lipid peroxidation, retard plant growth, and eventually cell death in apple as well as other fruit trees^6–9^. In addition, Cd may enter the human body via the edible parts of contaminated plants, posing a serious threat to human health^10^. Therefore, how to reduce Cd absorption and bioaccumulation and mitigate its toxicity in plants has attracted widespread attention^11,12^.

Grafting is the main way to propagate horticultural crops and affects plant tolerance to various stresses, including drought^13^, salinity^14^, and heavy metals (HM)^15^. Certain rootstocks can protect scions from HM toxicity by inhibiting or restricting HM ion transport^16^. A grafting experiment showed that the proper rootstock can drastically reduce the Cd content in the leaves of tomato and eggplant scions and improve plant nutritional balance^17^. However, HM uptake and tolerance were also determined by the scion in grafted combination. For example, significant differences in Cd bioaccumulation, translocation, and tolerance were observed in Hanfu and Fuji apples grafted onto the same rootstocks, *Malus baccata* or *M. micromalus* “qingzhoulinqin”^18^. Furthermore, a reciprocal grafting experiment demonstrated that Zn hypertolerance of *Thlaspi caerulescens* was primarily driven by the shoot^19^. Scion genotype is an important factor to alleviate aluminium-induced photosynthesis reduction in grafted citrus^20^. Therefore, the appearance of grafting *Malus* plants in HM bioaccumulation and tolerance was determined by their rootstock, scion, and their interaction. However, the prospective relationship between rootstock and scion that regulates HM bioaccumulation and detoxification at the transcriptional level remains largely unknown.

Apple (*M. domestica*) is an important economic fruit in the world, ranking first in yield and cultivation area in China^21,22^. However, mining, vehicle exhaust, and the unreasonable application of metal-based pesticides and fertilisers have caused Cd bioaccumulation in apple orchard soils^23,24^. Our previous studies showed differences between the Cd bioaccumulation, translocation, and tolerance among apple rootstocks of *M. baccata* and *M. micromalus* “qingzhoulinqin”^25,26^. Compared with *M. baccata*, *M. micromalus* “qingzhoulinqin” had a high ratio of Cd absorption and transport ability, making it more susceptible to oxidative stress^25,26^. Hanfu apple is a variety with cold and drought resistance and high yield, and has become the main variety grown in the cool areas of the Northeast. Previous results showed that Hanfu and Fuji apples had significant differences in cold resistance^27^. Further research showed that Hanfu and Fuji apple scion grafted onto either *M. baccata* or *M. micromalus* “qingzhoulinqin” had significant differences in Cd bioaccumulation, translocation, and tolerance^18^, which suggested that the Cd uptake, translocation, and detoxification of the *Malus* grafting plant is affected by their rootstock, scion, and their interaction. Thus, the above graft combinations are ideal materials for exploring how rootstock–scion interaction affects *Malus* transcriptome profiles in response to Cd.

In this study, fine roots of rootstock and scion leaves were taken as test materials for transcriptional reprogramming to explore the complete transcriptome profiles of grafted plant response to a Cd environment. In grafted plants, roots touch and absorb soil ions^28^, and the leaves play an important role in the distribution of heavy metals^29,30^. The gene expression differences among the four grafting combinations may be due to the scion, rootstock, or their interaction. Thus, this study includes pivotal tissue response of grafted combinations to a Cd environment. In our dataset, conserved gene clusters in rootstock and scion are observed to identify gene families involved in Cd uptake, transport, or other important functions in plants. For example, we analyse differentially expressed genes (DEGs) for Cd uptake and transport in rootstock roots and scion leaves of grafted combinations.

Weighted gene correlation network analysis showed that roots and leaves were enriched in plant hormone signal transduction and plant-pathogen interaction (data not shown). The genes of starch and sucrose metabolism in leaves were highly expressed, which enhanced the tolerance of grafted seeding to Cd. This difference and the crosstalk between tissues in the Cd absorption, transport, and detoxification of grafted combinations are worthy of in-depth analysis. In addition, the study of rootstock, scion, and their interaction can be used to assist genetic markers for plants with heavy metal tolerance. Although the mechanism of Cd response in individual tissues of plants has been explored, our dataset explored the underlying mechanisms by which rootstock, scion, and their interaction regulate the response of grafted plants to Cd stress. Based on this, we believe that this dataset will provide new insights for parsing Cd tolerance in fruit trees. The study of different apple grafted plants can be used to compare the variation of adaptive traits and provide a research basis for efficient and high-quality apple cultivation.

## Methods

### Plant material

Hanfu (HF) and Fuji (FJ) apple (*M. domestica*) cultivars grafted onto apple rootstocks of *M. baccata* (Mb) or *M. micromalus* “qingzhoulinqin” (Mm) were used as materials. Seeds of Mb and Mm were stratified by sand at 0–4 °C for 65 d. In early April 2019, germinated seeds were sown in nursery plates for 45 d in a greenhouse under the following growth conditions: 26 °C/18 °C day/night temperature and 50–60% humidity. Seedlings of uniform growth performance were planted in plastic pots using sand as the substrate and irrigated with 50 mL half-strength Hoagland nutrient solution^31^ (pH 6.0) every 2 d. In April 2020, virus-free HF and FJ budwoods were grafted onto rootstocks of Mb or Mm with uniform diameters (4–5 mm). After 12 weeks of bud germination, plants with similar growth status were transplanted into Hoagland nutrient solution, which was renewed every 2 d (pH 6.0).

### Cd experiment

**Fig. 1:**
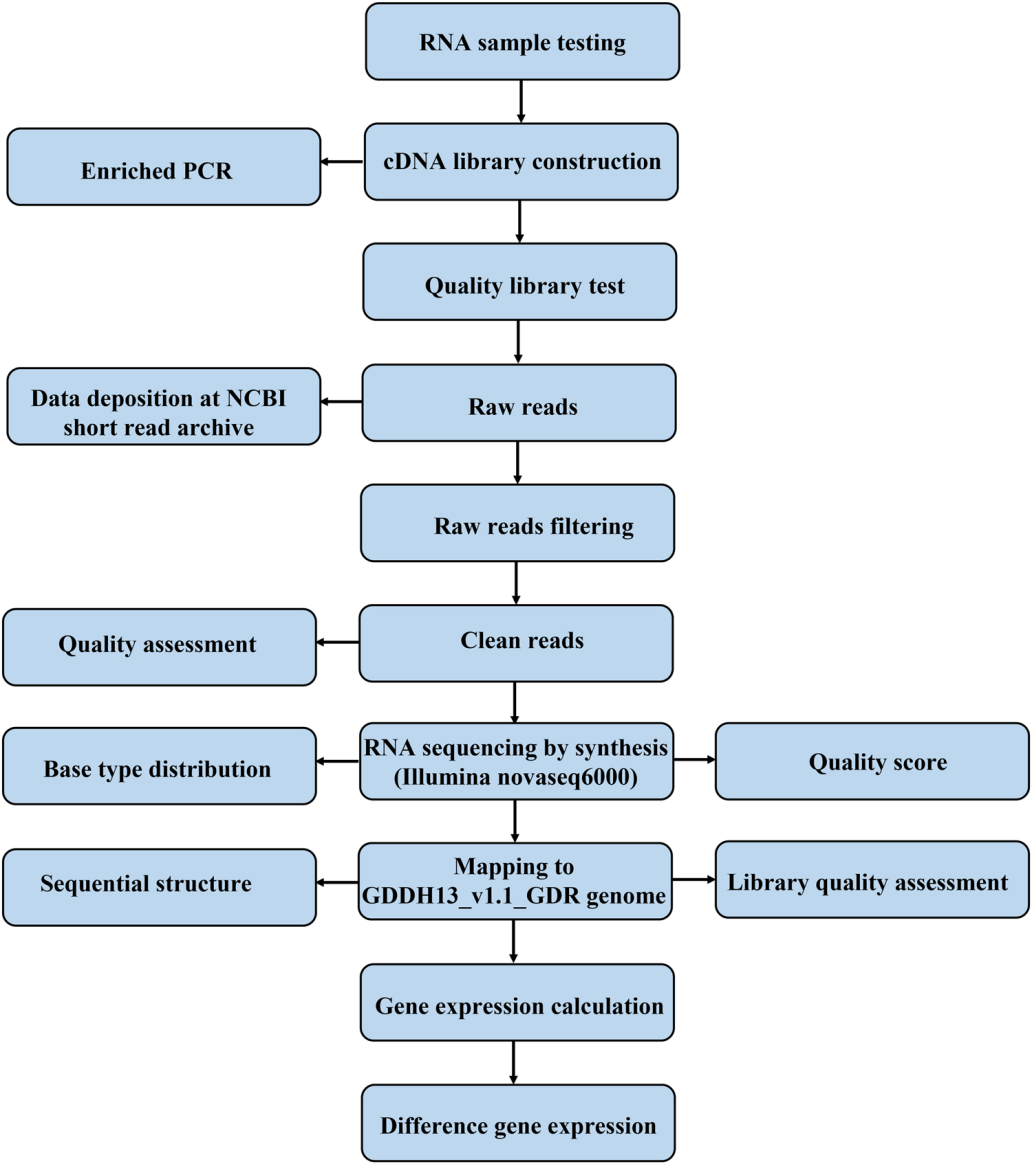
Flow chart of transcriptome data acquisition from RNA sample acquisition.

After 14 d of growth, 144 plants with similar growth status from four graft combinations were divided into two groups. Eighteen plants were cultured in Hoagland solution containing either 0 or 50 μM Cd^2+^, and three replicates for each graft combination under control or excess Cd stress were conducted, with each replicate containing six plants. After 70 d of Cd treatment, the roots and leaves of grafted plants were harvested separately. Cd^2+^ and reagent residues on the root surface were carefully rinsed with 20 mM EDTA disodium solution and deionised water. After the water on the root surface was wiped off, samples were long-term stored at −80 °C. Samples of roots and leaves were milled to a fine powder under liquid nitrogen maintenance and used to extract RNA. The procedure of RNA testing and acquisition was depicted in Fig. 1.

### RNA extraction, library preparation and sequencing

Total RNA was extracted from homogenised samples of the roots and leaves of four grafted plants. RNA extraction was performed with the method described by CLB + RN40-EASYspin (Aidlab Biotechnologies Co., Ltd, Beijing, China). Library was constructed with ≥1 μg of total RNA with excellent quality and integrity. Libraries were processed with Illumina for cluster generation on the flowcell and sequenced in 150 bp double-end mode on the Illumina novaseq 6000 (Illumina, San Diego, CA, USA).

### Validation of transcriptome by quantitative RT-PCR

The concentration and integrity of RNA samples returned by Biomarker Technologies was determined by spectrophotometer (NanoDrop^TM^ 2000, Thermo Fisher Scientific, Waltham, MA, USA) and agarose gel electrophoresis, respectively. The experiment of RNA reverse transcription was performed according to PrimeScript RT reagent Kit with gDNA Eraser (DRR037A, Takara, Dalian, China). Nine DEGs were randomly selected for quantitative real time polymerase chain reaction (qRT-PCR) with SYBR Green Premix Ex Taq II (DRR820A, Takara, Dalian, China). The specifically primers for each gene shown in Table S1 and the CFX96 real time system (CFX96, BioRad, Hercules, CA, USA) was applied to test relative gene expression. *β-Actin* was selected as the internal control gene and three replicates were performed for each sample. Relative expression levels were calculated according to the 2^−ΔΔCt^ method^32^.

## Data Records

Raw data of RNA-seq analysis were deposited in the Sequence Read Archive (SRA) of the National Center for Biotechnology Information (NCBI) under the accession number SRP424534^33^. Tables for differentially expressed genes in the roots and leaves of four grafted combinations are available under 10.6084/m9.figshare.22815575.v1^34^.

## Technical Validation

An Agilent 2100 Bioanalyzer (Agilent Technologies, Santa Clara, CA, USA) was used to determine RNA integrity. The RNA integrity numbers (RIN) were 8.47 ± 0.12 for the fine root and 8.23 ± 0.03 for leaf samples.

### Quality assessment

**Fig. 2:**
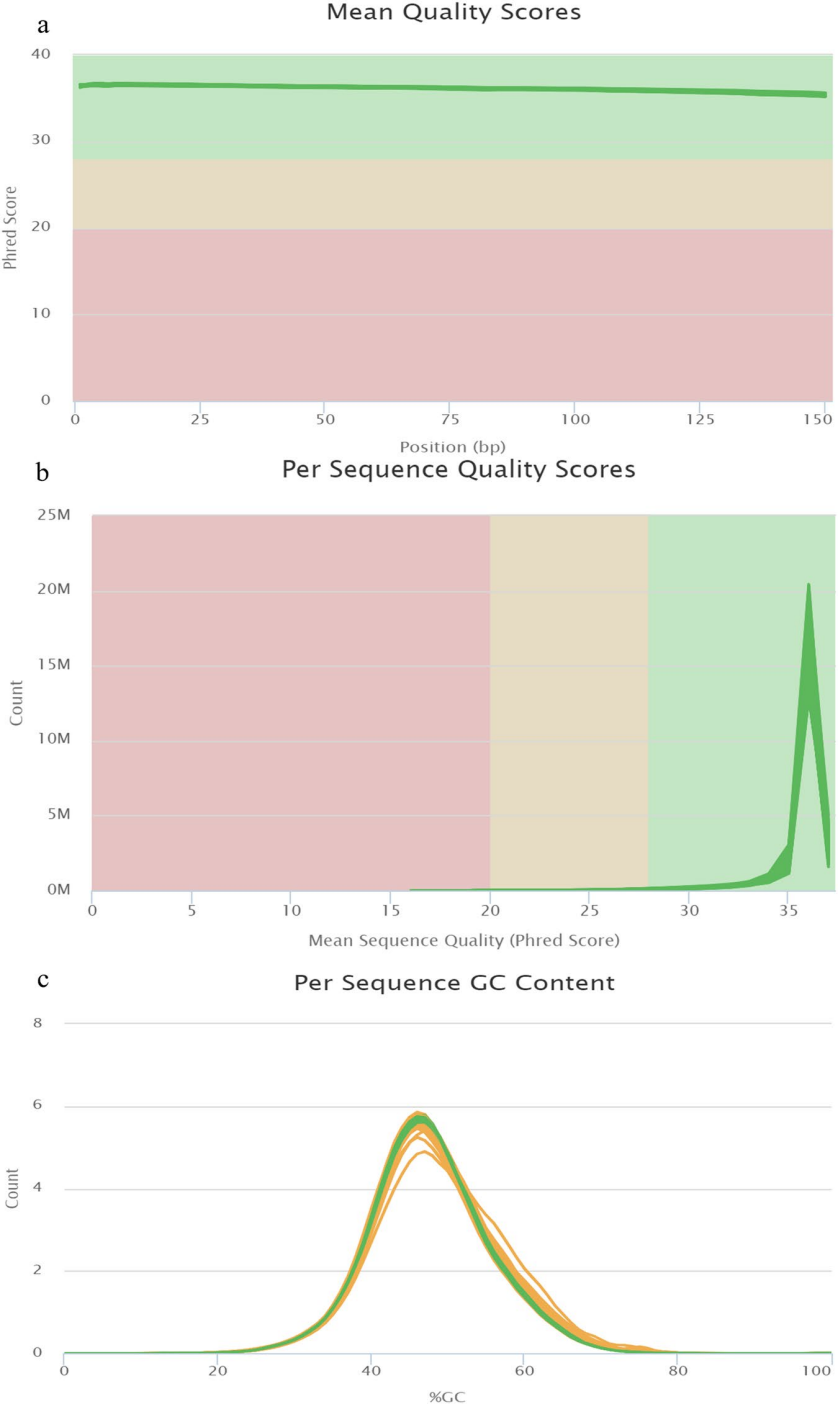
Quality assessment of RNA sequencing data. (**a**) Mean quality scores per sample; (**b**) per sequence quality scores of individual samples; (**c**) GC content per sample.

Based on sequencing by synthesis (SBS) technology, the Illumina Nova6000 high-throughput sequencing platform was used to perform transcriptome sequencing of the cDNA library. The transcriptome analysis was completed on 48 samples, and the clean data of each sample reached 5.71 Gb. FastQC was used as a quality control tool for high throughput sequencing data. The experimental results showed that the quality score of all sequences was > 30 (Fig. 2a) and the per-sequence quality scores were largely concentrated in the range of 30–40 (Fig. 2b), indicating that the base error rate was < 0.03% and the reads were of high quality. The GC distribution of all sequences was normally distributed and close to the theoretical distribution shape, which meant the read information had high quality (Fig. 2c).

### Data filtering and processing

The Python package Cutadapt v1.4.2 was used to access the raw sequence reads. Fastp (q 15 -u 50 -y -g -Y 10 -e 20 -l 100 -b 150 -B 150) was used to filter connectors and low-quality reads. Low-quality reads were removed, which were those with a proportion of N > 10% or the quality value Q ≤ 10 accounting for > 50% of the reads. mRNA was set to 0.1, used soap to align reads (soap -a 1.fq -b 2.fq -D /share/nas2/database/sRNA_database/current/ncRNA_integer.fasta.index -o out.pe −2 out.se -m 100 -x 1000 -u unmap.fa). HISAT2 (--dta -p 6 --max-intronlen 50) was used to align the trimmed reads with the *Malus domestica* GDDH13_v1.1_GDR transcriptome database^35^. A count table was generated using StringTie 1.3.4d (--merge -F 0.1 -T 0.1)^36^.

### Principal component analysis

**Fig. 3:**
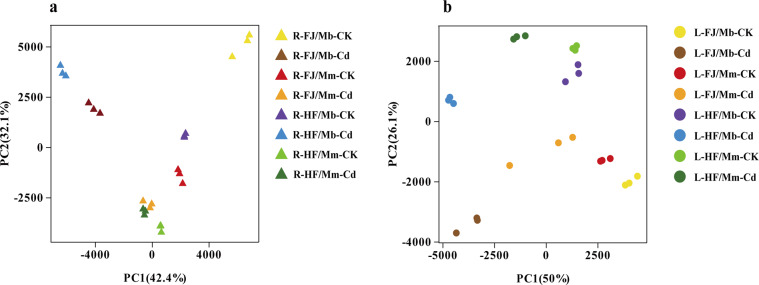
PCA of RNA-seq count data. (**a**) PCA of roots; (**b**) PCA of leaves.

Principal component analysis (PCA) classified the RNA-seq data from different treatments and tissues, which provided a foundation for data exploration. PCA results were extracted and visualised using ggplot2^37^. Four grafted combinations were separated from each other after Cd exposure, suggesting that Cd affected the transcript abundance of the roots and leaves of grafted combinations (Fig. 3). Irrespective of Cd treatment, a strong separation according to species (Mb and Mm) or cultivars (FJ and HF) was observed in root and leaf tissues, suggesting a strong variation of transcript abundance among species or cultivars. In addition, the leaves of the same apple cultivar scions grafted onto different rootstocks (Mb or Mm) were separated, and a similar result was found in the roots (Fig. 3). These results indicate that gene expression patterns in a graft combination are affected by rootstock, scion, and their interaction.

### Initial analysis of differentially expressed genes

**Fig. 4:**
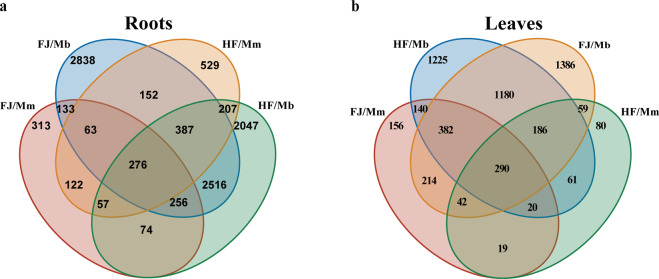
Venn diagram of DEGs in roots and leaves of different *Malus* grafted combinations under 0 or 50 μM CdCl_2_. (**a**) Intersections of Cd-regulated DEGs in root tissues of grafted combinations; (**b**) intersections of Cd-regulated DEGs in leaf tissues of grafted combination.

R package DESeq 2 1.6.3 (default: test = “Wald”, fitType = “parametric”) was applied to analyse the count data^38^. Genes with library sizes aligned one read per million reads were retained in reproducible samples for each treatment group. Venn diagrams were constructed using an online Venn diagram tool (https://jvenn.toulouse.inrae.fr/app/example.html). DEGs were tissue specific after 50 μM Cd exposure (Fig. 4). The number of DEGs in roots of grafted combinations was greater than that in leaves exposed to Cd. In the four grafted combinations, 276 shared genes were found in the roots, while 290 shared genes were observed in the leaves (Fig. 4a,b). The number of roots specific DEGs in the scions (FJ and HF) grafted onto Mb rootstock was greater than those grafted onto Mm, 42.9% in FJ/Mb, 35.2% in HF/Mb, 24.2% in FJ/Mm, and 29.5% in HF/Mm (Fig. 4a). These results indicated that Mb and Mm are two rootstock genotypes with different responses to Cd conditions and Mb is more susceptible (Fig. 4a). Moreover, specific DEGs were found between FJ/Mb and HF/Mb, and FJ/Mm and HF/Mm, indicating that gene expression patterns in the roots were affected by the scions. In leaves tissues, the largest number of specific DEGs was found in FJ/Mb (37.1%), followed by HF/Mb (35.2%), FJ/Mm (12.4%), and HF/Mm (10.6%), revealing that scion cultivars affect the gene expression patterns of leaves in response to Cd stress (Fig. 4b). The proportion of specific DEGs of leaves in the scions grafted onto Mb was approximately 3.0 times that of those grafted onto Mm, indicating that the rootstock also influences gene expression patterns in the leaves. These results reveal that the gene expression patterns of *Malus* grafted combinations to Cd stress are affected by rootstock, scion, and their interaction. This initial analysis shows this dataset can be applied to identify conserved processes of Cd absorption, transport, and detoxification that are shared by all four grafting combinations, as well as the specific Cd responses in *Malus* plants regulated by rootstock, scion, and their interaction.

### qRT-PCR vertification of RNA-seq data

**Fig. 5:**
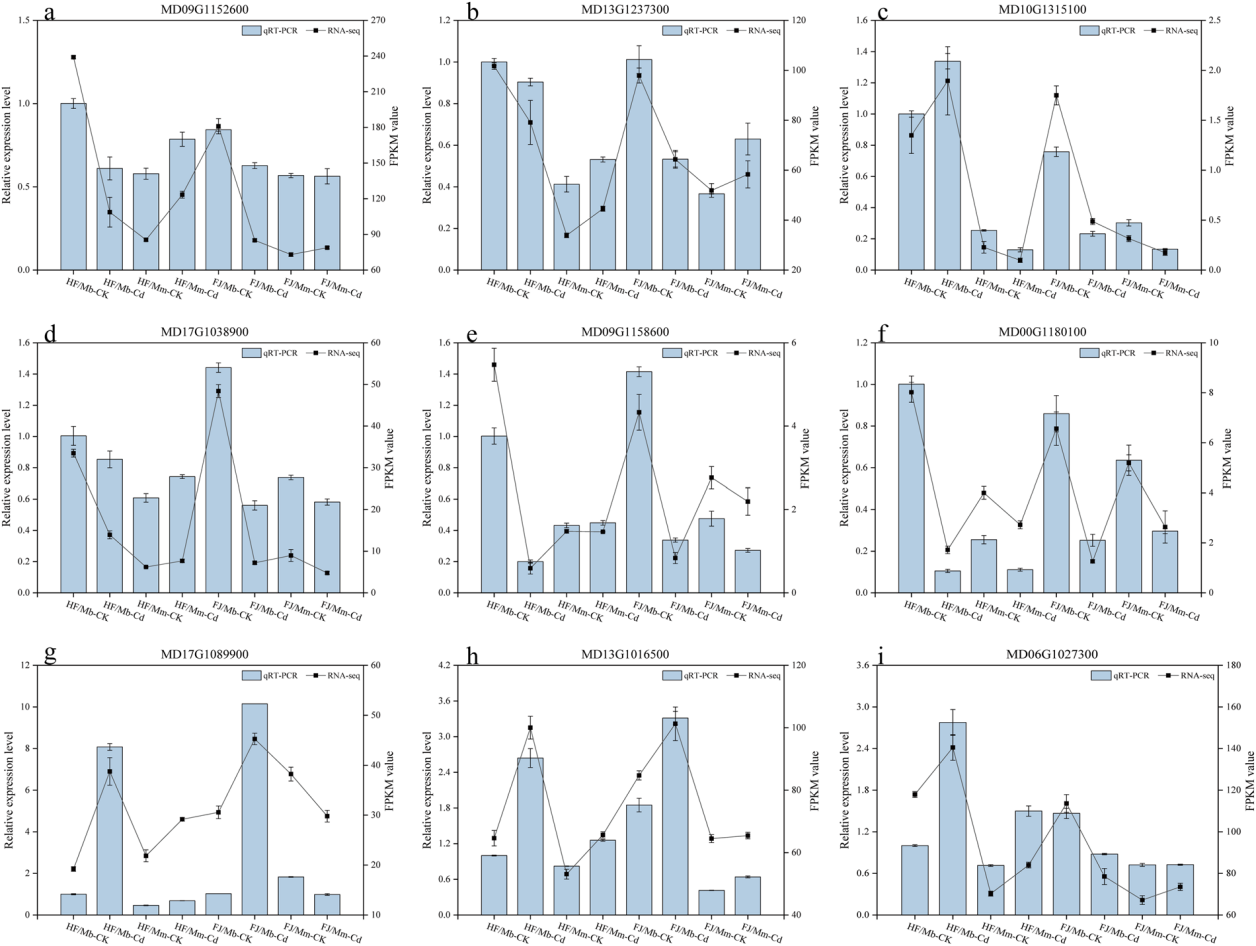
Relative gene expression of nine DEGs in the roots by qRT-PCR and RNA sequencing.

The results of qRT-PCR of nine selected genes were in accordance with transcriptome profiles, indicating the validity of RNA-seq results (Fig. 5).

## Supplementary information

Supplementary Information Table S1

## Data Availability

PCA analysis: R prcomp in software (3.1.1). Differential analysis: R package DESeq 2 1.6.3. Veen: https://jvenn.toulouse.inrae.fr/app/example.html.
