# Supplementary material for: Rootstock–scion interaction affects Malus transcriptome profiles in response to cadmium
Source: Sci Data. 2023 May 23;10:312. doi: 10.1038/s41597-023-02239-3 (PMC10205808; doi:10.1038/s41597-023-02239-3)
Supplement: Supplementary file 1 — Supplementary Information Table S1 [file 41597_2023_2239_MOESM1_ESM.docx]

**Rootstock–scion interaction affects *Malus* transcriptome profiles in response to cadmium**

Yijin Huang^1^, Luyang Sun^1^, Jiale Wang^1^, Yahui Chen^1^, Jiali He^1, 2*^, Deguo Lyu^1, 2^

1. College of Horticulture, Shenyang Agricultural University, Shenyang, Liaoning 110866, China

2. Key Lab of Fruit Quality Development and Regulation of Liaoning Province, Shenyang, Liaoning 110866, China

Corresponding author: Jiali He (hejiali1017@163.com; hejiali1017@syau.edu.cn)

Target journal: *Scientific Data*

**Supplementary Information**

**Table S1. Gene ID and primers used for qRT-PCR.**

| **Gene ID** | **Gene name** | **Primer-Forward** | **Primer-Reverse** |
| --- | --- | --- | --- |
| **MD09G1152600** | ***XTH1*** | **TACTGCACTGACCGAGCCCGAT** | **TCTCTGTCCCTCTGGCACTCTGGT** |
| **MD13G1237300** | ***XTH2*** | **TCAGGCACTTCCCGTTGTCCTT** | **GCCCTGTGGAATCGAGTGACTGA** |
| **MD10G1315100** | ***XTH3*** | **TCCCCAATGAGAAGCCGATGTACTT** | **GCCCATTCGTCTGCGTTCCATA** |
| **MD17G1038900** | ***XTH4*** | **GTCCCATACCCAAAGCTCCAACCTA** | **CCACAACGTCGAAAACACTCCCA** |
| **MD09G1158600** | ***XTH5*** | **CCACCAGAACCACCAGCTCGTT** | **GCGGAATCGGTGCAGTAGTCGTA** |
| **MD00G1180100** | ***XTH8*** | **GCAAAGGCAACAGAGAGCAGCAA** | **GGAAGTCGGCAGTTGGGTCAAA** |
| **MD17G1089900** | ***XTH9*** | **GACCCGACAGCAGTACAGGACCAT** | **TCTTGGGGTCCCTGCAATAGTCAT** |
| **MD13G1016500** | ***XTH10*** | **CAATCCCCAAGAAGCTGAAAGACTG** | **TCTGCTCCGGTGGTGATGCTT** |
| **MD06G1027300** | ***XTH11*** | **GCAGCCACAAACGACAGTCCAA** | **CTCGGTTGCGTTTGTGGGACTT** |
